# Zfp281 Inhibits the Pluripotent-to-Totipotent State Transition in Mouse Embryonic Stem Cells

**DOI:** 10.3389/fcell.2022.879428

**Published:** 2022-05-20

**Authors:** Xinpeng Wen, Zesong Lin, Hao Wu, Lanrui Cao, Xudong Fu

**Affiliations:** ^1^ Center of Stem Cell and Regenerative Medicine and Bone Marrow Transplantation Center of the First Affiliated Hospital, Zhejiang University School of Medicine, Hangzhou, China; ^2^ Zhejiang Laboratory for Systems and Precision Medicine, Zhejiang University Medical Center, Hangzhou, China; ^3^ Institute of Hematology, Zhejiang University, Hangzhou, China

**Keywords:** totipotency, pluripotency, Zfp281, Tet1, 2C-like cells, primed-state pluripotency

## Abstract

The cell-fate transition between pluripotent and totipotent states determines embryonic development and the first cell-lineage segregation. However, limited by the scarcity of totipotent embryos, regulators on this transition remain largely elusive. A novel model to study the transition has been recently established, named the 2-cell-like (2C-like) model. The 2C-like cells are rare totipotent-like cells in the mouse embryonic stem cell (mESC) culture. Pluripotent mESCs can spontaneously transit into and out of the 2C-like state. We previously dissected the transcriptional roadmap of the transition. In this study, we revealed that Zfp281 is a novel regulator for the pluripotent-to-totipotent transition in mESCs. Zfp281 is a transcriptional factor involved in the cell-fate transition. Our study shows that Zfp281 represses transcripts upregulated during the 2C-like transition *via* Tet1 and consequentially inhibits mESCs from transiting into the 2C-like state. Interestingly, we found that the inhibitory effect of Zfp281 on the 2C-like transition leads to an impaired 2C-like-transition ability in primed-state mESCs. Altogether, our study reveals a novel mediator for the pluripotent-to-totipotent state transition in mESCs and provides insights into the dynamic transcriptional control of the transition.

## Introduction

Totipotency refers to the ability of a cell to generate all cell types (Lu and Zhang, 2015). In mouse embryos, zygotes and 2-cell embryos are considered totipotent cells. When the embryo develops beyond zygotes and 2-cell (2C) stages, embryos progressively lose totipotency, go through the first lineage segregation, and establish pluripotent inner cell mass (ICM) at the blastocyst stage (Lu and Zhang, 2015). An impairment in the totipotent-to-pluripotent state transition in mouse embryos leads to defects in embryonic development, indicating that the transition is crucial for embryonic development (Percharde et al., 2018; Guo et al., 2019; Tian et al., 2020). However, limited by the material scarcity, mechanistic studies of the cell-fate transition between pluripotency and totipotency are largely impeded.

Mouse embryonic stem cells (mESCs) derived from the ICM were established as a model for pluripotency study (Li and Izpisua Belmonte, 2018). The mESC culture can be maintained in the ground-naïve, the metastable-naïve, or the primed state (Li and Izpisua Belmonte, 2018). When mESCs are cultured in the metastable-naïve condition, less than 1% of ESCs spontaneous transit into a totipotent-like state. This state, named as 2-cell-like (2C-like) state, exhibits several features of 2C-stage embryos, including totipotent-like developmental potential and the expression of 2C-specific transcripts, such as *Zscan4d* and MERVL repeats (Macfarlan et al., 2012; Fu et al., 2019).

The 2C-like transition is initiated by the transcription factor Dux. After Dux activation, pluripotent mESCs transit into the 2C-like state (the entry of 2C-like transition). The 2C-like state is unstable, and 2C-like cells can spontaneously transit back to the pluripotent state (the exit of the 2C-like transition). Notably, the exit of the 2C-like transition recapitulates the transcriptomic features of the transition from totipotency to pluripotency of embryonic development (Fu et al., 2020a; Fu et al., 2020b). Meanwhile, although the pluripotent-to-totipotent transition (the entry of 2C-like transition) does not exist in embryonic development, this transition serves as a valuable model for mechanistic investigation of totipotency (Percharde et al., 2018; Chen et al., 2021). Collectively, the 2C-like transition is currently a widely-used model for mechanistic exploration of cell-fate transition between totipotency and pluripotency (Boskovic et al., 2014; Dan et al., 2014; Lu et al., 2014; Ishiuchi et al., 2015; De Iaco et al., 2017; Hendrickson et al., 2017; Whiddon et al., 2017; Percharde et al., 2018; De Iaco et al., 2019; Eckersley-Maslin et al., 2019; Fu et al., 2019; Guo et al., 2019; Yan et al., 2019; Fu et al., 2020a; Fu et al., 2020b; Hu et al., 2020; Iturbide and Torres-Padilla, 2020; Qiu et al., 2020; Tian et al., 2020; Wu et al., 2020; Yang et al., 2020; Zhu et al., 2021a; Zhu et al., 2021b; Chen et al., 2021; Liu et al., 2021; Olbrich et al., 2021; Wang et al., 2021).

Our previous studies generated an inducible 2C-like transition model, a reporter mESC cell line containing a MERVL-promoter-driven reporter, and a doxycycline-inducible Dux transgene (synDux) (Fu et al., 2019). The synDux can drive the pluripotent-to-2C-like transition in mESCs, and the reporter can indicate whether the cells are in the 2C-like state. Importantly, synDux-induced 2C-like transition recapitulates the spontaneous 2C-like transition in the mESC culture (Fu et al., 2019; Fu et al., 2020a). Thus, this cell line is a valuable tool to monitor the entry and exit of the 2C-like transition. Using this model, we constructed the comprehensive roadmap for the 2C-like transition and revealed the regulatory network controlling the transition *via* a genome-wide CRISPR-Cas9-mediated screen (Fu et al., 2019; Fu et al., 2020a).

By examining the screen result, we identified that Zfp281 is a candidate factor affecting 2C-like transition. Zfp281 is a transcription factor modulating cell-fate transitions through transcriptional regulation. For instance, Zfp281 inhibits the expression of naïve-pluripotent-related genes *via* the interaction of Tet1 and consequentially promotes the naïve-to-prime transition in mESCs (Fidalgo et al., 2016). In addition, Zfp281 inhibits the transition from pluripotent cells to extraembryonic endoderm stem cells (XENs) by interacting with polycomb repressive complex 2 (PRC2) (Huang et al., 2021). Furthermore, Zfp281 promotes mESCs to transit into trophoblast stem cells (TSCs) *via* recruiting COMPASS (Complex Proteins Associated with Set1) to activate TSC-related genes (Ishiuchi et al., 2019). Taken together, these results suggest that Zfp281 plays a critical role in the cell-fate transition in mESCs. To this end, we set out to examine the function of Zfp281 in the 2C-like transition.

## Materials and Methods

### ESC Culture

The mESC-E14 cell line with MERV-L-LTR-tdTomato reporter was kindly provided by the laboratory of Jin Zhang from Zhejiang University. To generate the inducible 2C-like transition model, this reporter cell line was infected with lentivirus containing inducible Dux (addgene, cat. no. 138320), and clones were picked. The mESCs with the 2C::tdTomato reporter and doxycycline-inducible codon-optimized Dux cells were cultured on 0.1% gelatin-coated plates with standard leukemia inhibitory factor (LIF)/serum medium containing 10% FBS (HAKATA, cat. no. HB-FBS-500), 1,000 U/ml mouse LIF (Biolegend, cat. no. 554002), 0.1 mM non-essential amino acids (Gibco, cat. no. 11140), 0.055 mM β-mercaptoethanol (Gibco, cat. no. 21985023), 2 mM GlutaMAX (Gibco, cat. no. 35050), 1 mM sodium pyruvate (Gibco, cat. no. 11360) and penicillin/streptomycin (100 U/ml) (Gibco, cat. no. 15140) in a humidified 5% CO2 atmosphere at 37°C. For the culture of ESC lines, the medium was changed daily, and cells were routinely passaged every other day. For ground-naïve-state culture conditions, mESCs were cultured on 0.1% gelatin-coated plates with N2B27 medium, 1x N2 supplement, 1x B27 supplement, 2 mM GlutaMAX, 0.055 mM β-mercaptoethanol, 100 U/ml penicillin/streptomycin, Gsk3β inhibitor (CHIR99021, 3 μM), Mek inhibitor (PD0325901, 1 μM) and 1,000 U/ml mouse LIF. For primed-state culture condition, mESCs and were culture on 0.1% gelatin-coated plates with N2B27 medium supplemented with Activin A (20 ng/ml) and Fgf2 (12 ng/ml).

### FACS

Flow cytometry analysis was performed using the BD FACSAria Fusion SORP. Data and images were analyzed and generated using FlowJo (V10) software. The gating strategy was shown in FACS figures.

### RNA Isolation and qPCR

Cellular RNA was collected using the FastPure Cell/Tissue Total RNA Isolation Kit V2 (Vazyme, cat. no. RC112). Complementary DNA was generated using the HiScript II 1st Strand cDNA Synthesis Kit (+gDNA wiper) (Vazyme, cat. no. R212), and qRT-PCR was performed using the Taq Pro Universal SYBR qPCR Master Mix x (Vazyme, cat. no. Q712) on XN-1000V (ABI). Relative quantification was performed using the comparative CT method with normalization to *Gadph*. Primers and other oligos used in the qPCR are listed in Supplementary Tables S1–S3.

### Construction of siRNA and Cell Transfection

Two pairs of interference sequences that targeted mouse Tet1, Cxxc1, and Kmt2d mRNA were designed and synthesized using the siRNA online design program (Merck). The three interference sequences were Tet1-1 (5′-tgt​aga​cca​tca​ctg​ttc​gac-3′), Tet1-2 (5′-gag​att​aac​gct​gga​aca​ag-3′), Cxxc1-1 (5′-gtc​gca​aaa​ccg​gac​atc​aat​t-3′), Cxxc1-2 (5′-acg​agc​ttg​agg​cca​tca​ttc-3′), and Kmt2d-1 (5′-gtt​cat​cga​gtt​gcg​aca​taa-3′), Kmt2d-2 (5′-gtc​cta​taa​cca​gcg​gag​tct-3′). DNA oligos containing the target sequences were annealed and synthesized by T7 RNAi Transcription Kit (Vazyme, cat. no. TR102-01). Transfection was mediated by Lipo6000 transfection reagent (Beyotime, cat. no. C0526). The cells post transfection was then collected for other assays.

### CRISPR–Cas9

The gene knockdown by CRISPR–Cas9 was performed in our previous reports (Fu et al., 2019). The sgRNA sequences are listed in Supplementary Table S2. Lentivirus was produced using the psPAX2-PMD2. G system in 293T cells. The mESCs were infected with lentivirus for 48 h in a medium containing1 μg/ml Polybrene. After 2 days of infection, cells were cultured in a medium containing 1 μg/ml puromycin for another 8 days to select for infected cells.

### Western Blotting

Cellular protein was purified using RIPA Lysis Buffer with protease inhibitor. Western blotting was carried out with gradient gel (BCM Biotech, cat. no. P2012) with the following antibodies: Zfp281 (1:3,000, ABclonal, cat. no. A12650), GAPDH (1:20,000, ABclonal, cat. no. AC033), goat anti-rabbit IgG (H + L) secondary antibody, HRP (1:10,000, ABclonal, cat. no. AS014) and goat anti-mouse IgG (H + L) secondary antibody, HRP (1:10,000, ABclonal, cat. no. S003).

### Pseudo-Genome Preparation

As repeat elements tend to have multiple highly similar copies along the genome, it is relatively complex to accurately align them and estimate their expression. Hence, we created a repeat pseudo-genome. We used a slightly modified version of the RepEnrich (v0.1) (Criscione et al., 2014) software. Briefly, for each repetitive element subfamily, a pseudo-chromosome was created by concatenating all genomic instances of that subfamily along with their flanking genomics 15bp sequences and a 200bp spacer sequence (a sequence of Ns). The pseudo-genome was then indexed using STAR (v.2.5.2b) (Dobin et al., 2013), and the corresponding gtf and refFlat files were created using custom scripts and by considering each pseudo-chromosome as one gene.

### Sequencing Alignment for Coding Genes

Raw reads were first trimmed using Trimmomatic (v.0.36). Illumina sequence adaptors were removed, the leading and tailing low-quality base pairs (fewer than 3) were trimmed, and a 4-bp sliding window was used to scan the reads and trim when the window mean quality dropped below 15. Only reads having at least 50-bp were kept. The resulting reads were mapped to the mm10 genome using STAR (Dobin et al., 2013) (v.2.5.2b) with the following parameters: outSAMtype BAM SortedByCoordinate–outSAMunmapped Within–outFilterType BySJout -outSAMattributes NH HI AS NM MD -outFilterMultimapNmax 20 -outFilterMismatchNmax 999 -quantMode TranscriptomeSAM GeneCounts. The generated gene expression count files generated by STAR were then used for estimating gene expression.

### Sequencing Alignment for Repeats

Multi-mapped reads and reads mapping to intronic or intergenic regions were extracted and then mapped to the repeat pseudo-genome. First, the TagReadWithGeneExon command of the dropseq tools (v1.13) (Macosko et al., 2015) was used to tag the reads into utr, coding, intergenic and intronic reads using the bam tag “XF”. Multi-mapped reads, intergenic and intronic reads were extracted and mapped to the repeat pseudo-genome using STAR. The STAR read counts were used as an estimate of repeat expression.

### RNA-Seq Normalization

For each sample, the gene and repeat expression matrices were merged. Then the “Trimmed Mean of *M* values” normalization (TMM) method (Robinson et al., 2010) from the R/Bioconductor package edgeR package (v3.24.0) was used to calculate the normalized expression (McCarthy et al., 2012).

### Differential Gene Expression Analysis of Bulk RNA-Seq Data

The R/Bioconductor edgeR package (v3.24.0) (McCarthy et al., 2012) was used to detect the differentially expressed genes between the different samples using the generalized linear model-based method. Genes showing more than twofold expression change and an FDR<0.0001 were considered as differentially expressed.

### Functional Enrichment Analysis

Clusterprofiler was used to perform GO function enrichment and KEGG pathway annotation (Krämer et al., 2014). The associated GO and pathway enrichment plots were generated using the ggplot2 package (v3.1.0).

### Chip-Seq Data Analysis

The Chip-seq data were downloaded from GEO datasets, and below are the corresponding GEO Accession numbers: GSE81045 (Zfp281), GSE24843 (Tet1), GSE12721 (H2K119ub), GSE158460 (H3K9me3), GSE48519 (H3K4me1, H3K4me3). Raw reads were trimmed using Trimmomatic53 and then mapped to the mm10 genome using Bowtie254 (v2.2.9). Multi-mapped and unmapped, low-quality reads were removed using sambamba55 (0.6.6). Chip-seq peaks were determined by MACS (v2.0.10), and the peaks were visualized using IGV software.

## Results

### The Transcription Factor Zfp281 Inhibits the Pluripotent-to-2C-Like Transition

**FIGURE 1:**
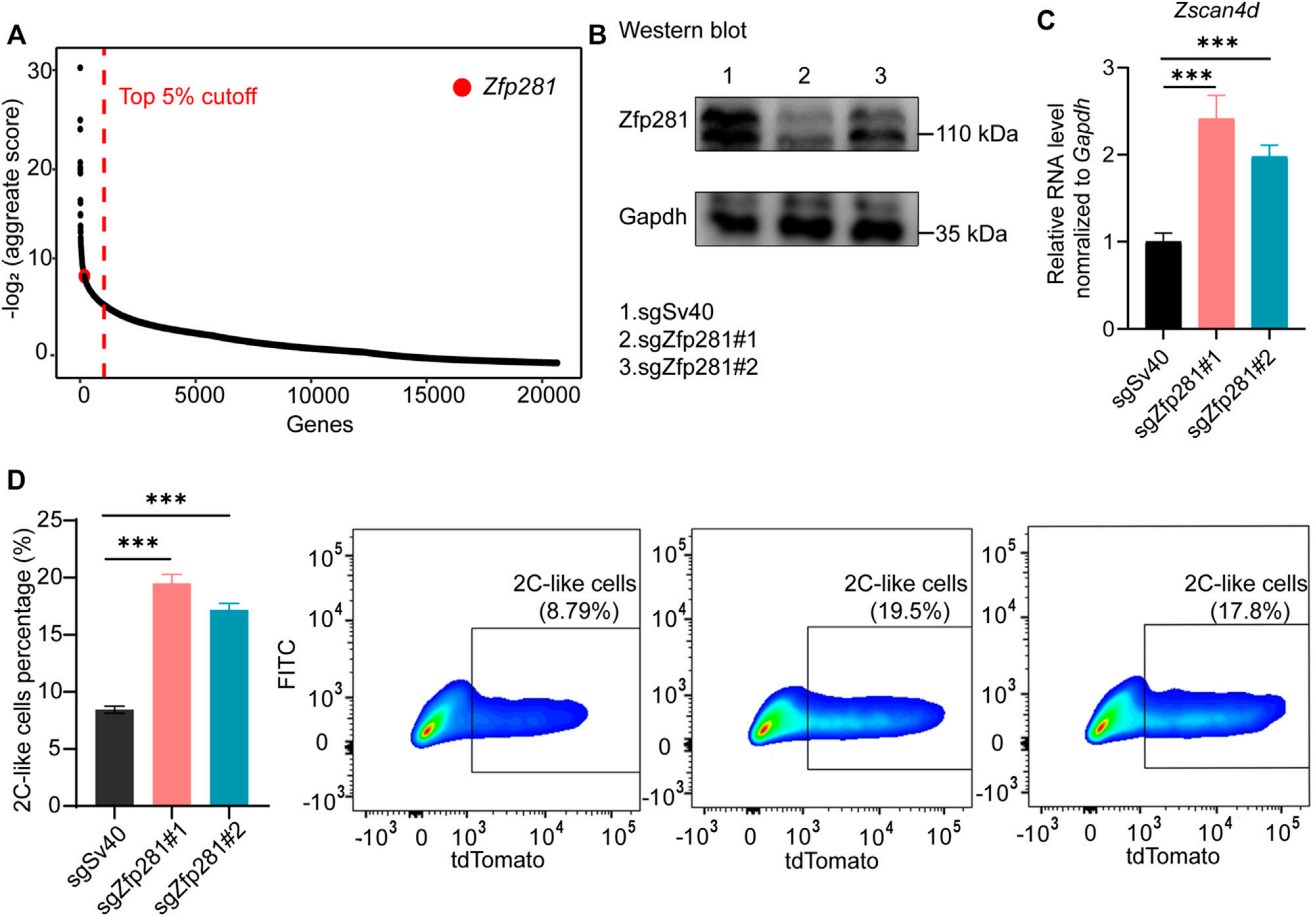
Zfp281 regulates the 2C-like transition of mESCs. **(A)** The sgRNA count enrichment from the published negative CRISPR-Cas9 genomic screen (Fu et al., 2019). Zfp281 is one of the top candidates inhibiting the 2C-like transition in the screen. **(B)** Western blot confirming efficient Zfp281 (bottom band) perturbation. sgZfp281#1 and sgZfp281#2 cause 67 and 38% decrease of Zfp281 expression respectively. **(C)** Relative *Zscan4d* mRNA levels normalized to *Gapdh* in *Dux*-activated mESCs. **(D)** The percentage of 2C-like cells after 24-hour *Dux* induction of the indicated manipulation in mESCs by FACS. **(B–D)** The X in sgX refers to the gene that sgRNA targets to; sgSv40 is negative control. Shown are mean ± s.d, *p* Values were calculated by unpaired t-test, two-tailed, two-sample unequal variance, *** <0.001.

We previously performed a genome-wide screen to search for factors mediating the 2C-like transition (Fu et al., 2019). Zfp281 is one of the top candidate factors inhibiting the 2C-like transition (Figure 1A). To validate the screen result, we used the inducible 2C-like transition model (Supplementary Figure S1A) to examine the role of Zfp281 on the 2C-like state. We design two independent sgRNA targeting Zfp281 and verify their efficiency (Figure 1B). Our results show that Zfp281 perturbation significantly increases the population of 2C-like cells and the expression of 2C-like-state marker genes after 24-h synDux induction (Figures 1C,D). Importantly, Zfp281 perturbation also increases the spontaneous 2C-like transition, and the expression of synDux is not altered upon Zfp281 perturbation (Supplementary Figures S1B,C), indicating that Zfp281 does not mediate 2C-like transition through synDux. In addition, Zfp281 does not bind to MERVL nor affect the expression of the MERVL-promoter-driven reporter (Supplementary Figures S1D,E), suggesting that Zfp281-perturbation does not directly regulate the reporter in our cell model. Lastly, Zfp281-knockdown by two independent siRNA significantly increases the expression of *Zscan4d* (2C-like-state marker gene) upon synDux induction (Supplementary Figure S1F) (Macfarlan et al., 2012). Altogether, these results suggest that Zfp281 regulates the 2C-like transition.

Zfp281 may affect the initiation, the entry, or the exit of the 2C-like transition. The initiation of the 2C-like transition is induced by Dux activation. We find that Zfp281-perturbation does not affect synDux or Dux expression (Supplementary Figures S1C,G), suggesting that the initiation of the transition is not affected by Zfp281. In addition, without initiating the 2C-like transition, Zfp281-perturbation exhibits minimal changes in the transcripts upregulated or downregulated during the 2C-like transition of metastable-naïve state mESCs (these transcripts are named as 2C-upregulated/downregulated transcripts, respectively in the following text, Supplementary Figure S1H and Supplementary Table S3). This result further suggests that Zfp281 does not affect the initiation of 2C-like transition. In addition, our results show that Zfp281-perturbed 2C-like cells exit from the 2C-like state at a similar rate compared to that of control cells (Supplementary Figure S1I), implying that Zfp281 does not modulate the exit of 2C-like transition. Notably, Zfp281-perturbation exhibits no effect on the cell growth within 48-h upon synDux induction, suggesting that Zfp281 does not mediate the transition through cell proliferation (Supplementary Figure S1J). Taken together, these results suggest that Zfp281 impedes the entry of 2C-like transition. Recently, novel totipotent reprogramming method, such as RNA-splicing inhibition, has been developed for mESCs. Our data shows that Zfp281-perturbation facilitates the activation of *Zscan4d* induced by the RNA-splicing inhibition (Supplementary Figure S1K), suggesting that Zfp281 is a general inhibitor for the pluripotent-to-totipotent state transition in mESCs.

### Zfp281 Inhibits the Expression of 2C-Upregulated Transcripts During the 2C-Like Transition

**FIGURE 2:**
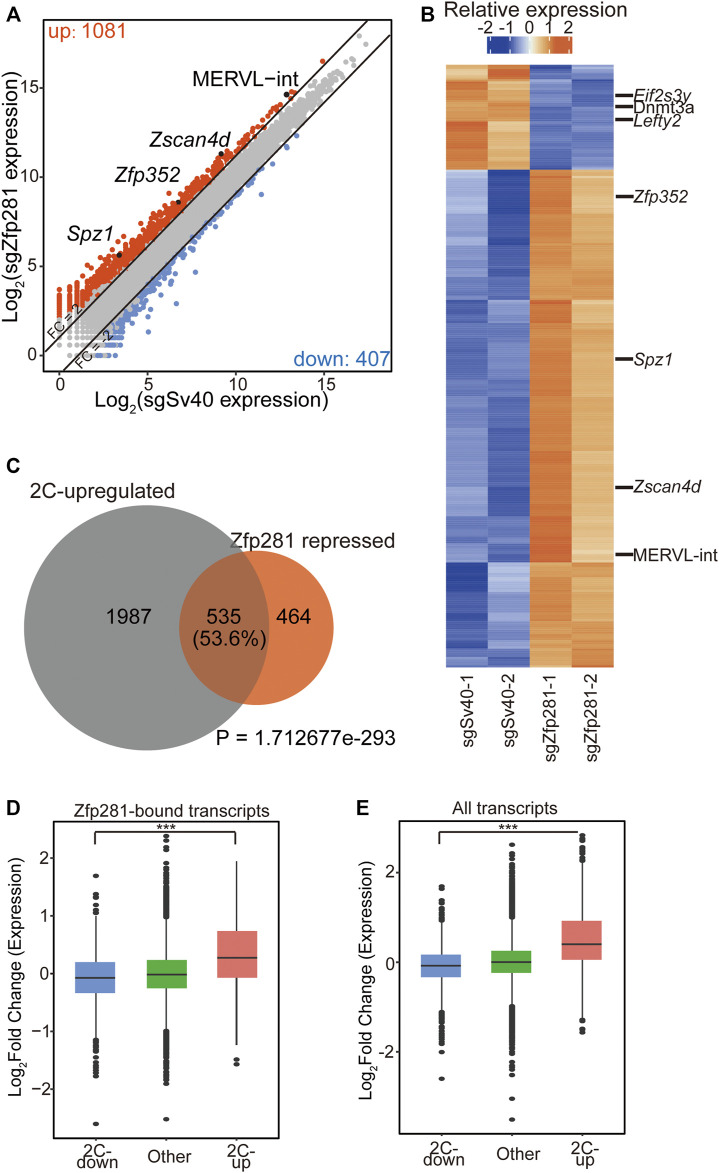
Zfp281 impedes the expression of 2C-upregulated transcripts during the 2C-like transition. **(A)** A scatter plot comparing the gene/repeat expression profiles between control and Zfp281-perturbed mESCs after *Dux* induction. The criteria for gene changes are Fold change (FC) > 2 and False discovery rate (FDR) < 0.05. **(B)** A heatmap showing the relative expression levels of differential expression genes/repeats (FC > 1, FDR < 0.05) from two biologically independent samples of control and Zfp281-perturbed mESCs after *Dux* induction. **(C)** A Venn diagram demonstrating overlaps of Zfp281-repressed transcripts and 2C-upregulated transcripts. Zfp281-repressed transcripts are defined as the transcripts activated in Zfp281-perturbed mESCs compared to control mESCs after *Dux* induction (FC > 2 and FDR < 0.05). *p* Value was calculated by a hypergeometric test. **(D)** A box plot showing the log_2_ (FC) of Zfp281-bound transcripts after *Dux* induction. Within all Zfp281-bound transcripts, 2C-upregulated transcripts exhibit the most significant changes. **(E)** A box plot showing the expression levels of 2C-regulated and other transcripts after *Dux* induction. **(A,B)** FDRs were estimated using the Benjamini–Hochberg method on the *p* Values of the two-sided quasi-likelihood F-test calculated using the edgeR package. **(D,E)** The black central line is the median value, the box limits indicate the upper and lower quartiles. **(A–E)** The X in sgX refers to the gene that sgRNA targets to; sgSv40 is negative control. **(D–E)** *p* Values were calculated with the absolute value of the log_2_ (FC) by the Wilcoxon rank-sum, *** <0.001. The dots represent outliers.

Previous studies indicated that Zfp281 regulated cell-fate transition through transcriptional regulation (Wang et al., 2008; Fidalgo et al., 2016; Dai et al., 2017; Ishiuchi et al., 2019; Luo et al., 2019; Huang et al., 2021). Thus, to understand how Zfp281 affects the 2C-like transition, we focus on the transcriptional effect of Zfp281 on the 2C-like transition. We perform RNA-seq on Zfp281-perturbed cells and control cells after 24-h synDux induction (Supplementary Figures S2A,B). By comparing the transcriptome, we identify 1,081 upregulated, and 407 downregulated transcripts upon Zfp281 perturbation (Figure 2A and Supplementary Table S3). The upregulated genes/repeats are Zfp281-repressed transcripts during the 2C-like transition. They include 2-cell-embryo-specific transcripts (Macfarlan et al., 2012) such as *Zscan4d, Zfp352*, and MERVL-int (Figure 2B), and the GO term of these transcripts is enriched in cell fate commitment (Supplementary Figure S2C). Importantly, most of these Zfp81-repressed transcripts are 2C-upregulated transcripts (Figure 2C), further supporting that Zfp281 impedes the pluripotent-to-2C-like transition. The downregulated genes include *Dnmt3a, Eif2s3y*, and *Lefty2* (Figure 2B) and are functionally enriched in Glycine, serine, and threonine metabolism (Supplementary Figure S2C).

To identify how Zfp281 shapes the transcriptome of 2C-like transition, we compare the published ChIP-seq results of Zfp281 with the RNA-seq data (Fidalgo et al., 2016). We find that although Zfp281 can bind to both 2C-downregulated and 2C-upregulated genes (Supplementary Figure S2D), the transcriptional changes of Zfp281-bound-2C-upregulated transcripts are more significant than that of Zfp281-bound-2C-downregulated transcripts (Figure 2D). Additionally, the change of total 2C-upregulated genes is significantly higher than that of total 2C-downregulated genes upon Zfp281 perturbation (Figure 2E). These results suggest that Zfp281 mainly regulates 2C-like transition by affecting 2C-upregulated genes. Previously, we identified that Myc majorly affects 2C-downregulated genes during the 2C-like transition. The transcriptional regulation of Zfp281 on 2C-like transition is different from that of Myc, and we find that knockdown of Myc can indeed further increases the transcriptional changes of 2C-like-state marker transcripts in Zfp281-perturbed cells (Supplementary Figure S2E). Interestingly, we find that Zfp281-unbound 2C-upregulated genes are significantly increased upon Zfp281 perturbation, implying that Zfp281 can shape the transcriptome of 2C-like transition both directly and indirectly (Supplementary Figure S2F).

### Tet1 Mediates the Inhibitory Effect of Zfp281 on the 2C-Upregulated Genes

Zfp281 shapes transcriptome through recruiting epigenetic elements to targeted genes (Wang et al., 2008; Fidalgo et al., 2016; Dai et al., 2017; Ishiuchi et al., 2019; Luo et al., 2019; Huang et al., 2021). For instance, Zfp281 inhibits the transcription of naïve-pluripotent genes by binding with Tet1 in mESCs (Fidalgo et al., 2016). To search for the factors that contributed to the effect of Zfp281 on 2C-upregulated transcripts, we analyze the ChIP-seq data of epigenetic factors reported to interact with Zfp281 (Ishiuchi et al., 2019; Dai et al., 2017). We find that Zfp281 colocalizes with H3k4me1, H3k4me3, and Tet1 in mESCs (Supplementary Figure S3A) but shows no colocalization of H2AK119ub and H3K9me3. Based on the ChIP-seq data, we chose two factors mediating H3K4 methylation (Cxxc1 and Kmt2d) and Tet1 for further study (Tate et al., 2010; Denissov et al., 2014; Sha et al., 2021).

Firstly, we focus on the methylation of H3K4. The knockdown of *Cxxc1* or *Kmt2d* significantly decreases H3K4me3 in mESCs (Supplementary Figure S3B). However, these manipulations do not consistently affect the expression of *Zscan4d* and MERVL upon Dux activation (Supplementary Figure S3C). These results indicate that the methylation of H3K4 does not affect 2C-upregulated genes during the 2C-like transition.

**FIGURE 3:**
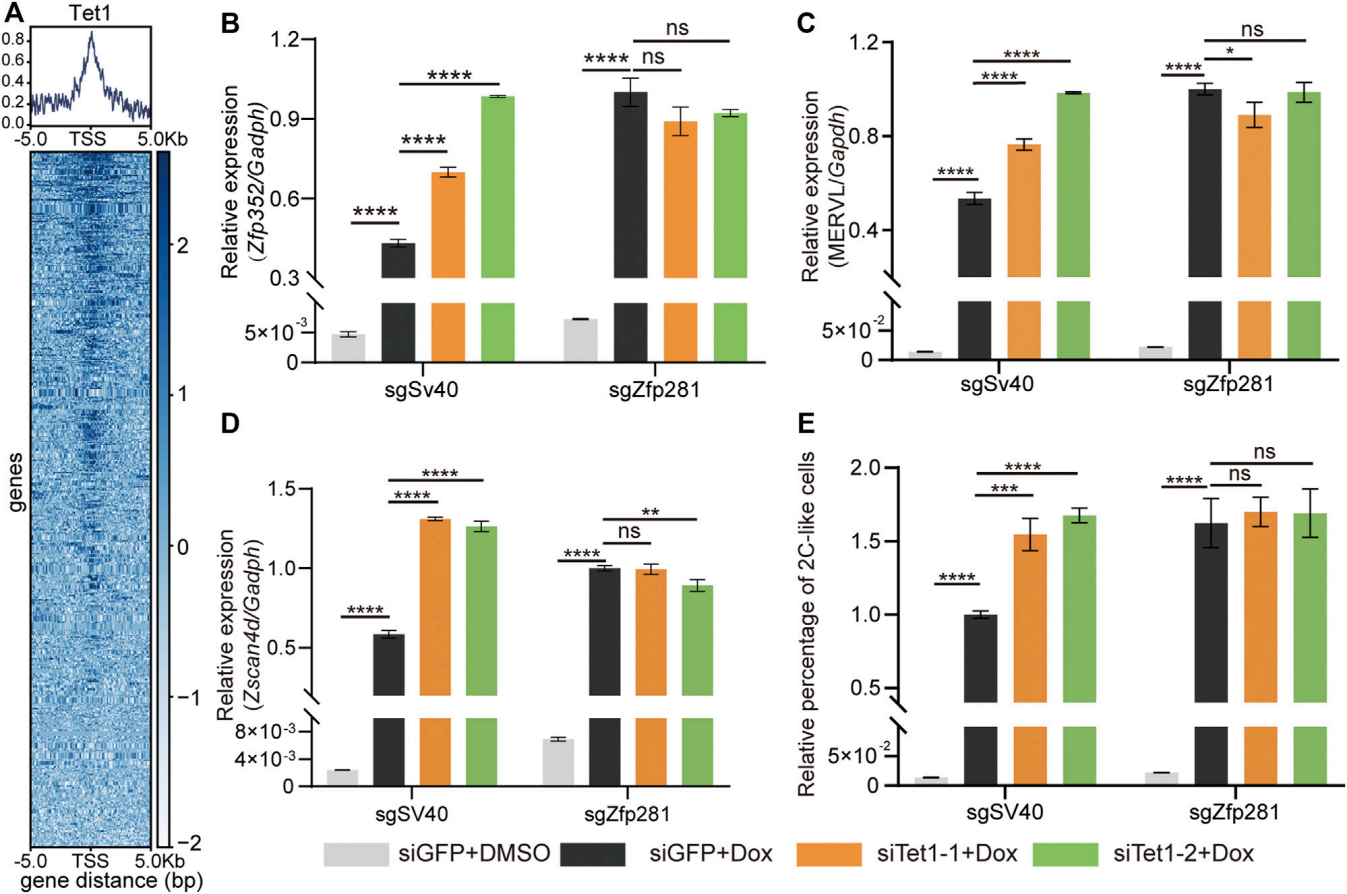
Zfp281 represses the 2C-like transition through Tet1. **(A)** Average occupancy plots and heatmaps of Tet1 signal within 5 kb of the center of TSS (transcription start sites) region of the Zfp281-bound-2C-upregulated genes. **(B–D)** Relative mRNA levels of *Zfp352* **(B)**, MERVL **(C)**, and *Zscan4d* **(D)** normalized to *Gadph* in mESCs upon indicated manipulation. Zfp281 majorly impedes the pluripotent-to-2C-like state transition (Figure 2). To accurately analyze the effect of Zfp281 on 2C-like transition, all results are analyzed at 12 h after synDux induction, when most mESCs are transiting into 2C-like state (Fu et al., 2019). **(E)** The relative percentage of 2C-like cells in mESCs upon indicated manipulation. **(B–E)** The siTet1-1/2 represents two independent siRNA targeting Tet1, siGFP is the negative control. Dox represents doxycycline. The X in sgX refers to the gene that sgRNA targets to; sgSv40 is negative control. Shown are mean ± s.d, *n* = 3. *p* Values were calculated by the parametric one-way analysis of variance (ANOVA) test, ns = no significance, ** <0.01, *** <0.001, **** <0.0001.

We next focus on Tet1. The major role of Tet1 is DNA demethylation (Wu et al., 2011). Interestingly, Tet1 plays a dual role in shaping the transcriptome of mESCs (Wu et al., 2011). It can activate and inhibit gene transcription by interacting with distinct epigenetic factors. Tet1 directly interacts with Zfp281 in mESCs (Fidalgo et al., 2016). In addition, the Tet family participates in the regulation of the 2C-like transition (Lu et al., 2014; Qiu et al., 2020; Huang et al., 2021). All these suggest that Tet1 contributes to the inhibitory effect of Zfp281 on 2C-upregulated transcripts during the 2C-like transition. To validate the hypothesis, we analyze the Tet1 and Zfp281 ChIP-seq data in mESCs. The majority of Zfp281-bound 2C-upregulated genes are bound by Tet1 (Figure 3A). Furthermore, within Zfp281-bound 2C-upregulated genes, the ones that are bound by Tet1 exhibit higher transcriptional changes than those not bound by Tet1 upon Zfp281 perturbation (Supplementary Figures S3D,E), suggesting that Tet1 mediates the inhibitory effect of Zfp281 on the 2C-upregulated genes.

Afterward, we test the transcriptional effects of Tet1 knockdown in Zfp281-perturbed and control mESCs (Supplementary Figure S3F). After *Dux* induction, Tet1 deficiency significantly increases the expression of 2C-like-state marker transcripts (*Zscan4d*, *Zfp352*, and MERVL) while showing no effect on these transcripts in Zfp281-perturbed cells (Figures 3B–D). These results suggest that Tet1 mediates the suppression effect of Zfp281 on 2C-upregulated genes during the 2C-like transition. Notably, Tet1 knockdown exhibits a larger effect than Zfp281-perturbation on *Zscan4d* (Figure 3D), indicating that Tet1 may inhibit the expression of *Zscan4d via* additional mechanisms other than Zfp281 (Lu et al., 2014). The diverse inhibitory effect of Tet1 on *Zscan4d* may contribute to the distinct genetic epistasis results of *Zscan4d* compared to that of MERVL and *Zfp352* (Figures 3B–D).

Lastly, we find that Tet1 knockdown significantly facilitates the 2C-like transition in control cells but does not affect the 2C-like transition in Zfp281-perturbed cells (Figure 3E). This result not only suggests that Zfp281 inhibits the 2C-like transition *via* Tet1 but also supports that Zfp281 mediates the 2C-like transition through transcriptional regulation.

### Zfp281 Contributes to the Impaired 2C-Like-Transition Ability in the Primed-State mESCs

The mESC can be cultured in three major pluripotent states, which are the ground-naïve state (cultured with MEK inhibitor, GSK3β inhibitors, and LIF, hereafter “2iL”), metastable-naïve state (cultured with serum and LIF, hereafter “SL”), and primed state (cultured with fibroblast growth factor 2 and Activin A, hereafter “FA”) (Fidalgo et al., 2016; Li and Izpisua Belmonte, 2018). The mESCs in the ground-naïve state exhibit significantly lower 2C-like transition compared to mESCs in the metastable-naive state (Macfarlan et al., 2012). One of the reasons is that ground-naïve mESC exhibits a higher expression of Nanog, which inhibits the 2C-like transition (Wu et al., 2020). The 2C-like transition in primed pluripotency has not been investigated. Interestingly, primed-state mESCs show higher Zfp281 expression compared to that of naïve-state mESCs (Fidalgo et al., 2016). Given that Zfp281 inhibits the 2C-like transition, we hypothesized that primed-state mESCs might exhibit decreased potential for the 2C-like transition.

**FIGURE 4:**
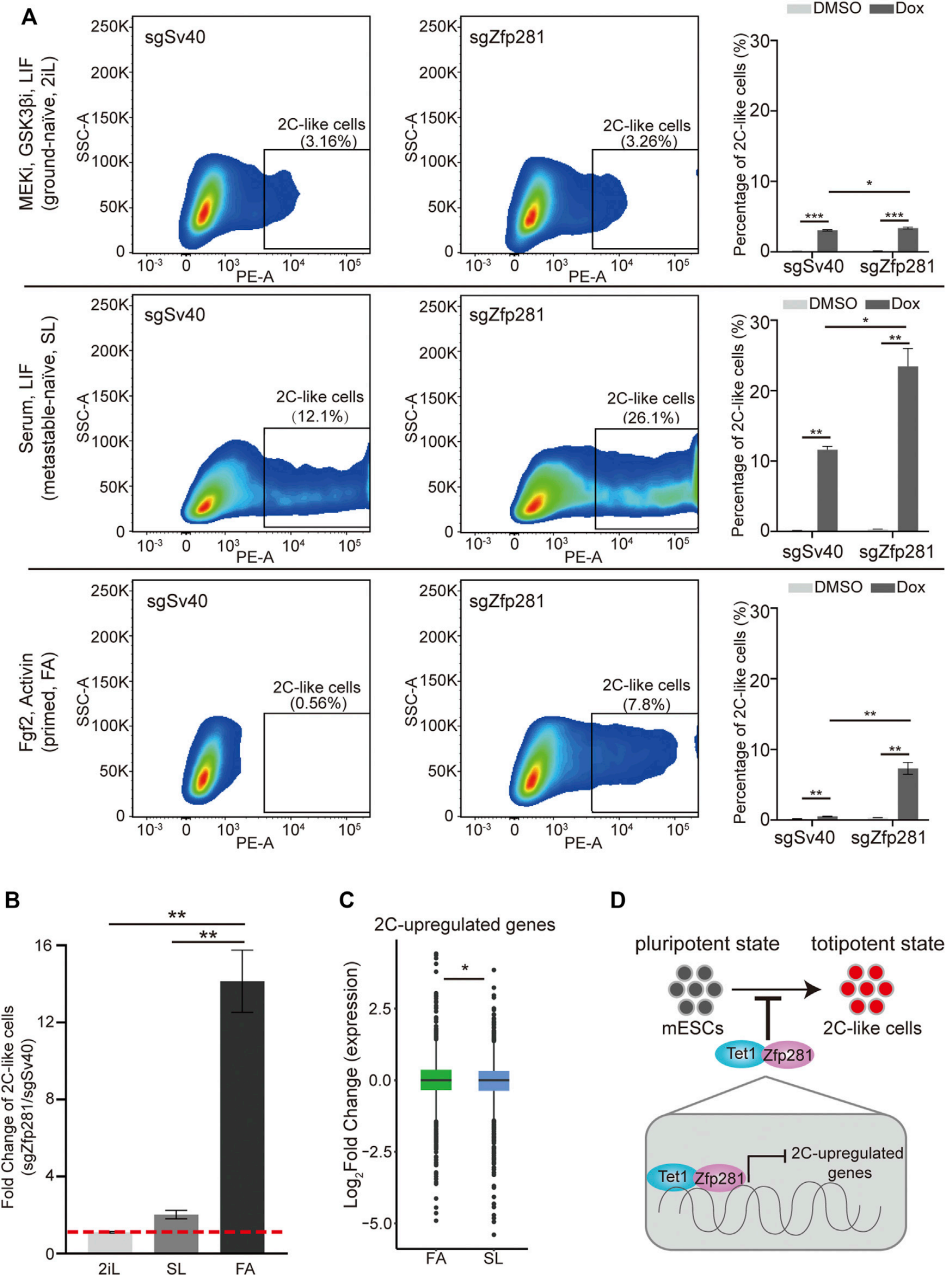
Zfp281 leads to an impaired 2C-like-transition ability in primed-state mESCs. **(A)** The percentage of 2C-like cell population in ground-naïve (2iL), metastable-naïve (SL), and primed states (FA) mESCs upon indicated perturbation. Representative FACS results are shown. **(B)** The fold change of the 2C-like cell population in three pluripotent states Zfp281-perturbed mESCs normalized to control mESCs. Redline indicates fold change of 1. **(A–B)** Shown are mean ± s.d, *n* = 3. *p* Values were calculated by unpaired t-test, two-tailed, two-sample unequal variance, * <0.05, ** <0.01, *** <0.0001. **(C)** A box plot demonstrating the log_2_ (fold change) of 2C-upregulated genes expression in metastable-naïve and primed states mESCs upon Zfp281 perturbation. The original RNA-seq was downloaded from GSE81045. *p* Values were calculated by the Wilcoxon rank-sum test, * <0.05. The dots represent outliers. **(D)** A model showing Zfp281 and Tet1 contributes to the impaired 2C-like-transition ability by repressing the expression of 2C-upregulated genes. **(A–C)** Dox represents doxycycline. The X in sgX refers to the gene that sgRNA targets to; sgSv40 is negative control.

To validate the hypothesis, we firstly compare the 2C-like transition ability of mESCs cultured in ground-naïve, metastable-naïve, and primed states. Consistent with previous results, ground-naïve state mESCs exhibit impaired 2C-like transition ability than mESCs cultured in the metastable-naïve state. Interestingly, primed-state mESCs exhibit an even lower ability of 2C-like transition compared to that of ground-naïve state mESCs (Figure 4A).

To test whether Zfp281 contributes to the decreased 2C-like transition in primed-state mESCs, we compare the effect of Zfp281-perturbation on 2C-like transition in mESCs maintained in different states. Although Zfp281 deficiency increases the 2C-like cells population in each pluripotent state (Figure 4A), the effects are distinct. The impact of Zfp281 on the 2C-like transition is most significant in primed-state mESCs (Figure 4B). The transcriptomic analysis also suggests that Zfp281-perturbation has a more substantial effect on 2C-upregulated transcripts in primed-state mESCs than in naïve-state mESCs (Figure 4C). On the contrary, Zfp281 exhibits a marginal impact on the 2C-like transition in ground-naïve-state mESCs (Figures 4A,B), which is consistent with the low expression of Zfp281 in ground-naïve state mESCs. These results indicate that Zfp281 contributes to the decreased 2C-like transition in primed-state mESCs but does not play a major role in the reduced 2C-like transition in ground-naïve-state mESCs.

## Discussion

The cell-fate transition between pluripotent and totipotent states is crucial for embryonic development. However, it is challenging to examine the transition due to the limited relevant biological materials. The 2C-like transition in mESCs has recently become a novel model to study the transition (Fu et al., 2020b; Iturbide and Torres-Padilla, 2020). In this study, by using an inducible 2C-like transition model, we revealed that Zfp281 impedes the pluripotent-to-2C-like transition (Figure 4D). Mechanistic-wise, we showed that Zfp281 inhibits the activation of 2C-upregulated genes through the interaction with Tet1.

Previous results indicate that Zfp281 plays an important role in mediating cell-fate-transition, including the transition between naïve and primed state pluripotency. Here, we revealed a novel function of Zfp281 in mediating pluripotent-to-2C-like state transition, further supporting that Zfp281 is a master regulator for cell-identity determination.

The 2C-like transition is initiated by the transcription factor Dux and is reversible. Our results indicate that Zfp281 specifically impedes the entry of 2C-like transition but exhibits no effect on the initiation or the exit of 2C-like transition. Notably, we found that Zfp281 shows no impact on the maintenance of the 2C-like state, indicating that Zfp281 is not required for the self-renewal of the 2C-like state.

Notably, Zfp281 does not inhibit the 2C-like transition by modulating the transcription of Dux or synDux (Supplementary Figures S1C,G). Instead, Zfp281 impedes the transition by inhibiting 2C-upregulated transcripts (Figure 2). Upon Dux or synDux induction, 2C-upregulated transcripts are transcriptionally activated, and the inhibitory effect of Zfp281 on these transcripts will become more prominent. Thus, Dux or synDux can amplify the inhibitory effect of Zfp281 on the 2C-like transition.

It has been reported that the Tet family inhibits the 2C-like transition by maintaining the expression of pluripotent genes, but the individual effect of the Tet family member on the 2C-like transition has not been carefully examined (Qiu et al., 2020). In this study, we showed that Tet1 interacts with Zfp281 and inhibits the 2C-like transition through impeding the activation of 2C-upregulated genes, indicating that the Tet family plays multi-dimensional roles in the pluripotent-to-totipotent transition. Notably, although it has been reported that Zfp281 directly interacts with Tet1 (Fidalgo et al., 2016), it is theoretically plausible that Tet1 and Zfp281 suppress 2C-like transition *via* redundant parallel pathways.

Lastly, our study compared the potential for the 2C-like transition in ground-naïve, metastable-naïve, and primed state pluripotent stem cells. Our results revealed that primed-state mESCs exhibit decreased potential for 2C-like transition, and Zfp281 contributes to the decrease. On the contrary, Zfp281 plays a minimal role in the 2C-like transition in ground-naïve-state mESCs, suggesting the role of Zfp281 on the 2C-like transition is dependent on the pluripotent state.

In conclusion, our study reveals the function of Zfp281 on the 2C-like transition and the underlying mechanisms. It is interesting to investigate whether Zfp281 plays a similar role in totipotent mouse embryos and human ESCs.

## Data Availability

The datasets presented in this study can be found in online repositories. The names of the repository/repositories and accession number(s) can be found in the article/Supplementary Material. The RNA-seq dataset generated during this study has been deposited to NCBI Gene Expression Omnibus (GEO, GSE201478).

## References

[B1] Boskovic A. Eid A. Pontabry J. Ishiuchi T. Spiegelhalter C. Raghu Ram E. V. (2014). Higher Chromatin Mobility Supports Totipotency and Precedes Pluripotency *In Vivo* . Genes Dev. 28, 1042. 10.1101/gad.238881.114 24831699 PMC4035533

[B2] Chen C. Liu W. Guo J. Liu Y. Liu X. Liu J. (2021). Nuclear m^6^A Reader YTHDC1 Regulates the Scaffold Function of LINE_1_ RNA in Mouse ESCs and Early Embryos. Protein Cell 12, 455–474. 10.1007/s13238-021-00837-8 33886094 PMC8160034

[B3] Criscione S. W. Zhang Y. Thompson W. Sedivy J. M. Neretti N. (2014). Transcriptional Landscape of Repetitive Elements in normal and Cancer Human Cells. BMC Genomics 15, 583. 10.1186/1471-2164-15-583 25012247 PMC4122776

[B4] Dai Q. Shen Y. Wang Y. Wang X. Francisco J. C. Luo Z. (2017). Striking a Balance: Regulation of Transposable Elements by Zfp281 and Mll2 in Mouse Embryonic Stem Cells. Nucleic Acids Res. 45, 12301–12310. 10.1093/nar/gkx841 29036642 PMC5716208

[B5] Dan J. Liu Y. Liu N. Chiourea M. Okuka M. Wu T. (2014). Rif1 Maintains Telomere Length Homeostasis of ESCs by Mediating Heterochromatin Silencing. Develop. Cel 29, 7–19. 10.1016/j.devcel.2014.03.004 PMC4720134 24735877

[B6] De Iaco A. Coudray A. Duc J. Trono D. (2019). DPPA_2_ and DPPA_4_ Are Necessary to Establish a 2C-like State in Mouse Embryonic Stem Cells. EMBO Rep. 20, 7382. 10.15252/embr.201847382 PMC6500978 30948459

[B7] De Iaco A. Planet E. Coluccio A. Verp S. Duc J. Trono D. (2017). DUX-family Transcription Factors Regulate Zygotic Genome Activation in Placental Mammals. Nat. Genet. 49, 941–945. 10.1038/ng.3858 28459456 PMC5446900

[B8] Denissov S. Hofemeister H. Marks H. Kranz A. Ciotta G. Singh S. (2014). Mll2 Is Required for H3K4 Trimethylation on Bivalent Promoters in Embryonic Stem Cells, whereas Mll1 Is Redundant. Development 141, 526–537. 10.1242/dev.102681 24423662

[B9] Dobin A. Davis C. A. Schlesinger F. Drenkow J. Zaleski C. Jha S. (2013). STAR: Ultrafast Universal RNA-Seq Aligner. Bioinformatics 29, 15–21. 10.1093/bioinformatics/bts635 23104886 PMC3530905

[B10] Eckersley-Maslin M. Alda-Catalinas C. Blotenburg M. Kreibich E. Krueger C. Reik W. (2019). Dppa2 and Dppa4 Directly Regulate the Dux-Driven Zygotic Transcriptional Program. Genes Dev. 33, 194–208. 10.1101/gad.321174.118 30692203 PMC6362816

[B11] Fidalgo M. Huang X. Guallar D. Sanchez-Priego C. Valdes V. J. Saunders A. (2016). Zfp281 Coordinates Opposing Functions of Tet1 and Tet2 in Pluripotent States. Cell Stem Cell 19, 355–369. 10.1016/j.stem.2016.05.025 27345836 PMC5010473

[B12] Fu X. Djekidel M. N. Zhang Y. (2020). A Transcriptional Roadmap for 2C-Like-To-Pluripotent State Transition. Sci. Adv. 6, eaay5181. 10.1126/sciadv.aay5181 32523982 PMC7259939

[B13] Fu X. Wu X. Djekidel M. N. Zhang Y. (2019). Myc and Dnmt1 Impede the Pluripotent to Totipotent State Transition in Embryonic Stem Cells. Nat. Cel Biol. 21, 835. 10.1038/s41556-019-0343-0 PMC7137718 31209294

[B14] Fu X. Zhang C. Zhang Y. (2020). Epigenetic Regulation of Mouse Preimplantation Embryo Development. Curr. Opin. Genet. Develop. 64, 13–20. 10.1016/j.gde.2020.05.015 PMC7641911 32563750

[B15] Guo M. Zhang Y. Zhou J. Bi Y. Xu J. Xu C. (2019). Precise Temporal Regulation of Dux Is Important for Embryo Development. Cell Res 29, 956–959. 10.1038/s41422-019-0238-4 31591446 PMC6889123

[B16] Hendrickson P. G. Doráis J. A. Grow E. J. Whiddon J. L. Lim J.-W. Wike C. L. (2017). Conserved Roles of Mouse DUX and Human DUX_4_ in Activating Cleavage-Stage Genes and MERVL/HERVL Retrotransposons. Nat. Genet. 49, 925–934. 10.1038/ng.3844 28459457 PMC5703070

[B17] Hu Z. Tan D. E. K. Chia G. Tan H. Leong H. F. Chen B. J. (2020). Maternal Factor NELFA Drives a 2C-like State in Mouse Embryonic Stem Cells. Nat. Cel Biol 22, 175–186. 10.1038/s41556-019-0453-8 31932739

[B18] Huang X. Bashkenova N. Yang J. Li D. Wang J. (2021). ZFP281 Recruits Polycomb Repressive Complex 2 to Restrict Extraembryonic Endoderm Potential in Safeguarding Embryonic Stem Cell Pluripotency. Protein Cell 12, 213–219. 10.1007/s13238-020-00775-x 32812113 PMC7895869

[B19] Ishiuchi T. Enriquez-Gasca R. Mizutani E. Bošković A. Ziegler-Birling C. Rodriguez-Terrones D. (2015). Early Embryonic-like Cells Are Induced by Downregulating Replication-dependent Chromatin Assembly. Nat. Struct. Mol. Biol. 22, 662–671. 10.1038/nsmb.3066 26237512

[B20] Ishiuchi T. Ohishi H. Sato T. Kamimura S. Yorino M. Abe S. (2019). Zfp281 Shapes the Transcriptome of Trophoblast Stem Cells and Is Essential for Placental Development. Cel Rep. 27, 1742–1754. 10.1016/j.celrep.2019.04.028 31067460

[B21] Iturbide A. Torres-Padilla M.-E. (2020). A Cell in Hand Is worth Two in the Embryo: Recent Advances in 2-cell like Cell Reprogramming. Curr. Opin. Genet. Develop. 64, 26–30. 10.1016/j.gde.2020.05.038 32599301

[B22] Krämer A. Green J. Pollard J. Jr. Tugendreich S. (2014). Causal Analysis Approaches in Ingenuity Pathway Analysis. Bioinformatics (Oxford, England) 30, 523–530. 10.1093/bioinformatics/btt703 PMC3928520 24336805

[B23] Li M. Izpisua Belmonte J. C. (2018). Deconstructing the Pluripotency Gene Regulatory Network. Nat. Cel Biol 20, 382–392. 10.1038/s41556-018-0067-6 PMC6620196 29593328

[B24] Liu J. Gao M. He J. Wu K. Lin S. Jin L. (2021). The RNA m^6^A Reader YTHDC1 Silences Retrotransposons and Guards ES Cell Identity. Nature 591, 322–326. 10.1038/s41586-021-03313-9 33658714

[B25] Lu F. Liu Y. Jiang L. Yamaguchi S. Zhang Y. (2014). Role of Tet Proteins in Enhancer Activity and Telomere Elongation. Genes Dev. 28, 2103–2119. 10.1101/gad.248005.114 25223896 PMC4180973

[B26] Lu F. Zhang Y. (2015). Cell Totipotency: Molecular Features, Induction, and Maintenance. Natl. Sci. Rev. 2, 217–225. 10.1093/nsr/nwv009 26114010 PMC4477869

[B27] Luo Z. Liu X. Xie H. Wang Y. Lin C. (2019). ZFP281 Recruits MYC to Active Promoters in Regulating Transcriptional Initiation and Elongation. Mol. Cel Biol 39, e00329–19. 10.1128/MCB.00329-19 PMC6879198 31570506

[B28] Macfarlan T. S. Gifford W. D. Driscoll S. Lettieri K. Rowe H. M. Bonanomi D. (2012). Embryonic Stem Cell Potency Fluctuates with Endogenous Retrovirus Activity. Nature 487, 57–63. 10.1038/nature11244 22722858 PMC3395470

[B29] Macosko E. Z. Basu A. Satija R. NemeshShekhar J. K. ShekharBialas K. I. Tirosh I. (2015). Highly Parallel Genome-wide Expression Profiling of Individual Cells Using Nanoliter Droplets. Cell 161, 1202–1214. 10.1016/j.cell.2015.05.002 26000488 PMC4481139

[B30] McCarthy D. J. Chen Y. Smyth G. K. (2012). Differential Expression Analysis of Multifactor RNA-Seq Experiments with Respect to Biological Variation. Nucleic Acids Res. 40, 4288–4297. 10.1093/nar/gks042 22287627 PMC3378882

[B31] Olbrich T. Vega-Sendino M. Tillo D. Wu W. Zolnerowich N. Pavani R. (2021). CTCF Is a Barrier for 2C-like Reprogramming. Nat. Commun. 12, 4856. 10.1038/s41467-021-25072-x 34381034 PMC8358036

[B32] Percharde M. Lin C.-J. Yin Y. Guan J. Peixoto G. A. Bulut-Karslioglu A. (2018). A LINE1-Nucleolin Partnership Regulates Early Development and ESC Identity. Cell 174, 391–405. 10.1016/j.cell.2018.05.043 29937225 PMC6046266

[B33] Qiu Q. Hu P. Qiu X. Govek K. W. Cámara P. G. Wu H. (2020). Massively Parallel and Time-Resolved RNA Sequencing in Single Cells with scNT-Seq. Nat. Methods 17, 991–1001. 10.1038/s41592-020-0935-4 32868927 PMC8103797

[B34] Robinson M. D. McCarthy D. J. Smyth G. K. (2010). EdgeR: a Bioconductor Package for Differential Expression Analysis of Digital Gene Expression Data. Bioinformatics 26, 139–140. 10.1093/bioinformatics/btp616 19910308 PMC2796818

[B35] Sha Q.-Q. Zhu Y.-Z. Xiang Y. Yu J.-L. Fan X.-Y. Li Y.-C. (2021). Role of CxxC-finger Protein 1 in Establishing Mouse Oocyte Epigenetic Landscapes. Nucleic Acids Res. 49, 2569–2582. 10.1093/nar/gkab107 33621320 PMC7969028

[B36] Tate C. M. Lee J.-H. Skalnik D. G. (2010). CXXC finger Protein 1 Restricts the Setd1A Histone H3K4 Methyltransferase Complex to Euchromatin. FEBS J. 277, 210–223. 10.1111/j.1742-4658.2009.07475.x 19951360 PMC2806598

[B37] Tian Q. Wang X.-f. Xie S.-m. Yin Y. Zhou L.-q. (2020). H3.3 Impedes Zygotic Transcriptional Program Activated by Dux. Biochem. biophysical Res. Commun. 522, 422–427. 10.1016/j.bbrc.2019.11.114 31767152

[B38] Wang Y. Na Q. Li X. Tee W. W. Wu B. Bao S. (2021). Retinoic Acid Induces NELFA‐mediated 2C‐like State of Mouse Embryonic Stem Cells Associates with Epigenetic Modifications and Metabolic Processes in Chemically Defined media. Cel Prolif. 54, e13049. 10.1111/cpr.13049 PMC8168409 33960560

[B39] Wang Z.-X. Teh C. H.-L. Chan C. M.-Y. Chu C. Rossbach M. Kunarso G. (2008). The Transcription Factor Zfp281 Controls Embryonic Stem Cell Pluripotency by Direct Activation and Repression of Target Genes. Stem Cells 26, 2791–2799. 10.1634/stemcells.2008-0443 18757296

[B40] Whiddon J. L. Langford A. T. Wong C.-J. Zhong J. W. Tapscott S. J. (2017). Conservation and Innovation in the DUX4-Family Gene Network. Nat. Genet. 49, 935–940. 10.1038/ng.3846 28459454 PMC5446306

[B41] Wu H. D’Alessio A. C. Ito S. Xia K. Wang Z. Cui K. (2011). Dual Functions of Tet1 in Transcriptional Regulation in Mouse Embryonic Stem Cells. Nature 473, 389–393. 10.1038/nature09934 21451524 PMC3539771

[B42] Wu K. Liu H. Wang Y. He J. Xu S. Chen Y. (2020). SETDB1-Mediated Cell Fate Transition between 2C-like and Pluripotent States. Cel Rep. 30, 25–36. 10.1016/j.celrep.2019.12.010 31914391

[B43] Yan Y.-L. Zhang C. Hao J. Wang X.-L. Ming J. Mi L. (2019). DPPA2/4 and SUMO E3 Ligase PIAS4 Opposingly Regulate Zygotic Transcriptional Program. Plos Biol. 17, e3000324. 10.1371/journal.pbio.3000324 31226106 PMC6608977

[B44] Yang F. Huang X. Zang R. Chen J. Fidalgo M. Sanchez-Priego C. (2020). DUX-miR-344-ZMYM2-Mediated Activation of MERVL LTRs Induces a Totipotent 2C-like State. Cell Stem Cell 26, 234–250. 10.1016/j.stem.2020.01.004 32032525 PMC8074926

[B45] Zhu Y. Cheng C. Chen L. Zhang L. Pan H. Hou L. (2021). Cell Cycle Heterogeneity Directs Spontaneous 2C State Entry and Exit in Mouse Embryonic Stem Cells. Stem Cel Rep. 16, 2659–2673. 10.1016/j.stemcr.2021.09.003 PMC8580870 34624246

[B46] Zhu Y. Yu J. Gu J. Xue C. Zhang L. Chen J. (2021). Relaxed 3D Genome Conformation Facilitates the Pluripotent to Totipotent-like State Transition in Embryonic Stem Cells. Nucleic Acids Res. 49, 12167–12177. 10.1093/nar/gkab1069 34791385 PMC8643704

