# Supplementary material for: Zfp281 Inhibits the Pluripotent-to-Totipotent State Transition in Mouse Embryonic Stem Cells
Source: Front Cell Dev Biol. 2022 May 20;10:879428. doi: 10.3389/fcell.2022.879428 (PMC9163740; doi:10.3389/fcell.2022.879428)
Supplement: Supplementary file 1 [file DataSheet1.PDF]

Supplementary Material

Supplementary Figures

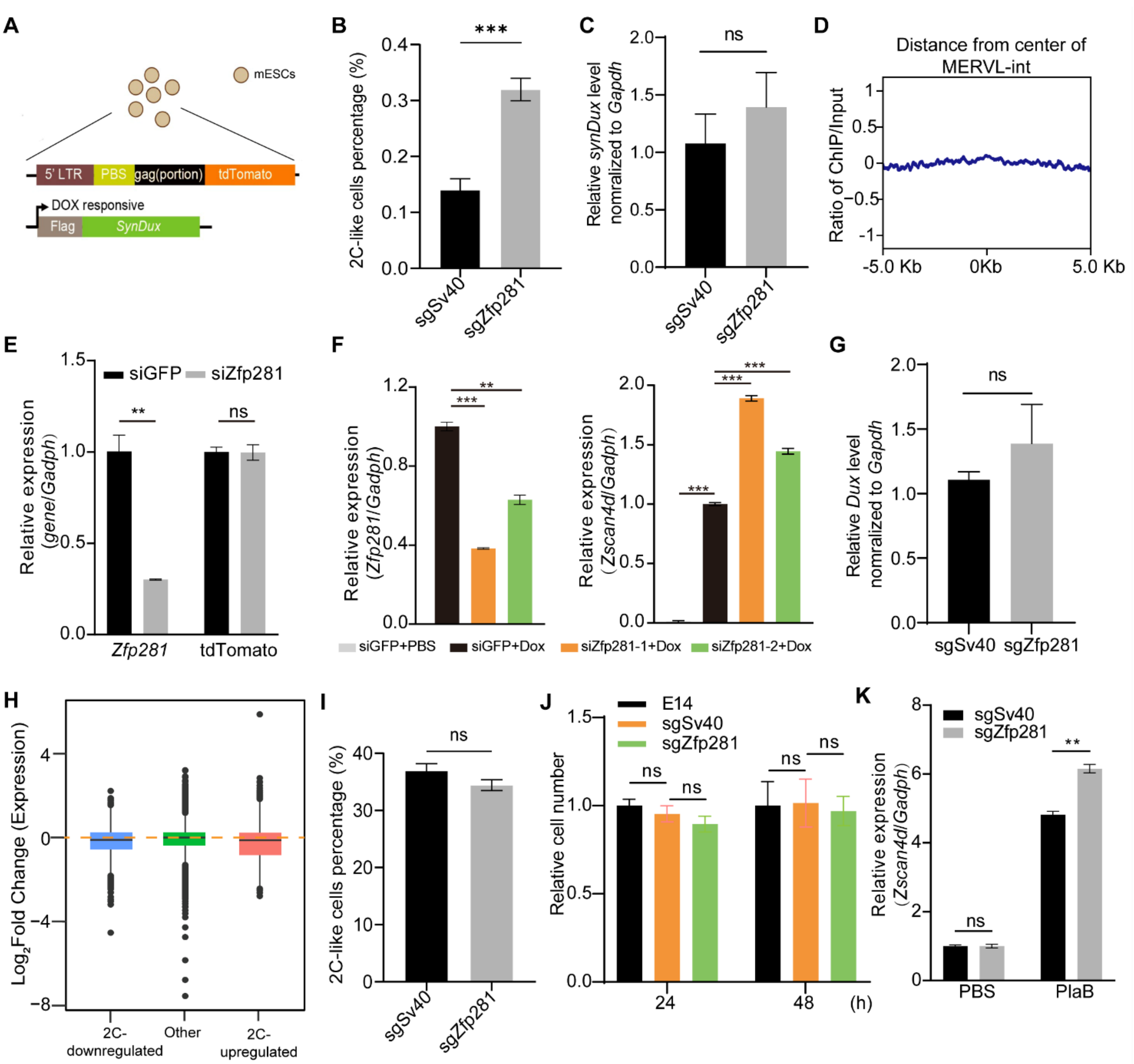

Fig. S1, Zfp281 inhibits the pluripotent-to-2C-like state transition in mESCs. (A) A schematic representation of the constructs in the mESCs. The tdTomato reporter is under the control of the MERVL promoter. syn*Dux* refers to codon-optimized exogenous *Dux*. PBS, primer binding site. LTR, long terminal repeats. The MERVL promoter consists of 5' LTR, PBS, and a portion of the MERVL gag gene. (B) The percentage of spontaneous 2C-like cells (2C-like cells without synDux induction) of the indicated manipulation in three independent mESCs by FACS. (C) Relative *synDux* mRNA levels normalized to *Gapdh* in Dux-activated mESCs. (D) Zfp281 binding profile plot around the center of MERVL-int sequence. The ChIP-seq signal was calculated as the ratio of normalized reads relative

to input. (E) The knockdown of Zfp281 in MEF cells does not affect the expression of the MERVL-promoter-driven tdTomato reporter. (F) Relative gene expression normalized to *Gapdh* upon indicated manipulation. MERVL-promoter-driven tdTomato reporter. (G) Relative *Dux* mRNA levels normalized to *Gapdh* in Dux-activated mESCs. (H) A box plot showing the $\log_2$(fold change) of 2C-regulated and other genes in Zfp281-perturbed normalized to control mESCs before *Dux* induction. The black central line is the median, the box limits indicate the upper and lower quartiles. The dots represent outliers. The orange dashed line indicates a fold change of 1. (I) The percentage of the 2C-like cells after 24-hour culture of isolated 2C-like cells. (J) The cell number of indicated cell lines upon 24 or 48-hour synDux activation. (K) Relative *Zscan4d* level normalized to *Gapdh* upon indicated treatment. Pladienolide B (PlaB, an inhibitor for RNA splicing) was used at 50nM for 24-hour. (A-K) Doxycycline (Dox) was used at 2μg/μl; the X in sgX refers to the gene that sgRNA targets to; sgSv40 is negative control. Shown are mean ± s.d, n = 3. P values were calculated by unpaired t-test, two-tailed, two-sample unequal variance, ns = no significance, *** < 0.001.

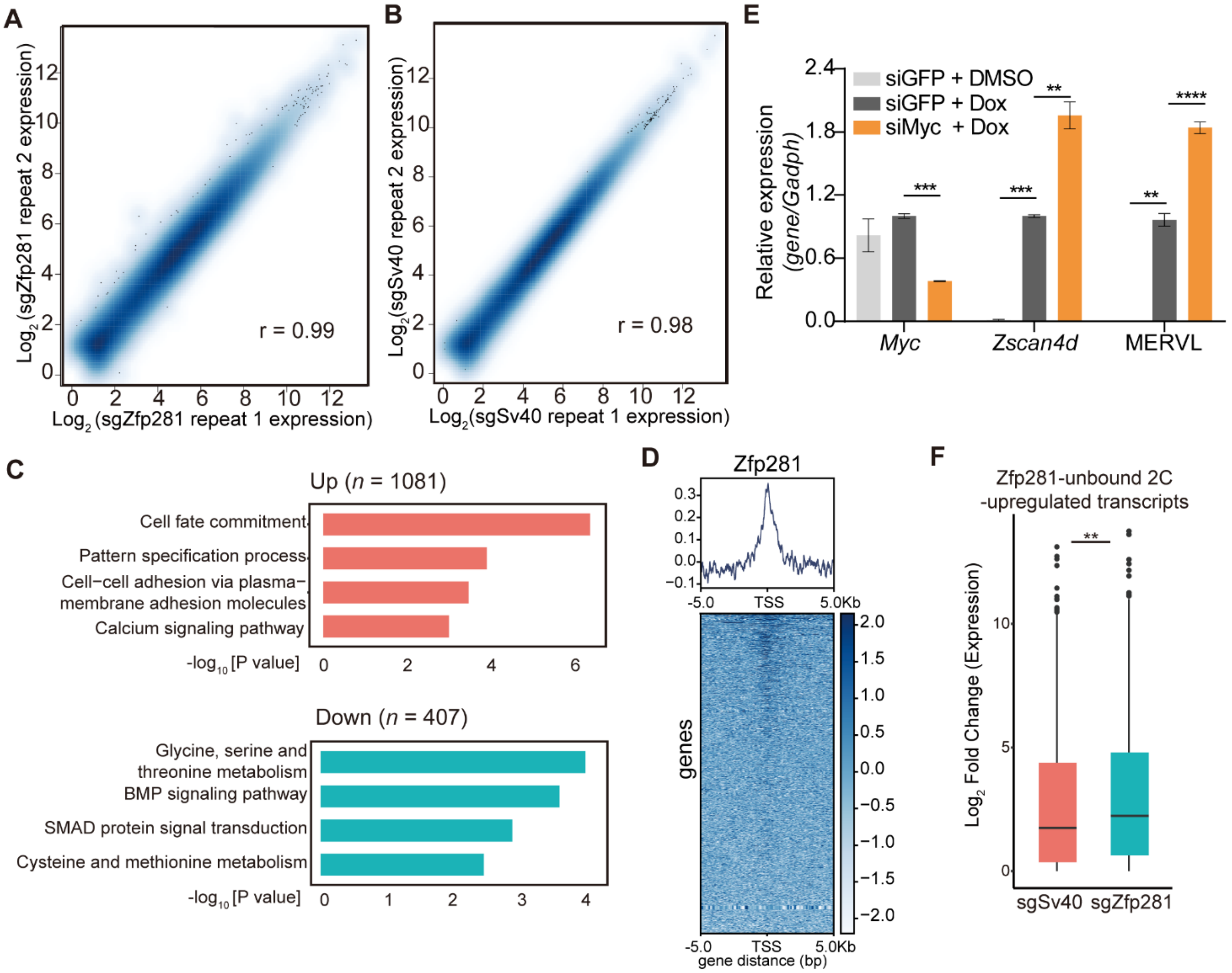

Fig. S2, Zfp281 regulates the transcriptome of the 2C-like transition. (A) Scatter plot comparing the gene expression profiles between two biologically independent samples of *Dux*-activated Zfp281-perturbed mESCs (Pearson correlation, r = 0.99). (B) Scatter plot comparing the gene expression profiles between two biologically independent samples of *Dux*-activated control mESCs (Pearson correlation, r = 0.98). (C) Bar plot showing the $-\log_{10}$ [P value] of the gene ontology (GO) terms enriched in each category of genes (right, right-tailed Fisher's exact test). The number of genes in each category is indicated at the top of each GO enrichment plot. (D) Average occupancy plots and heatmaps of Zfp281 signal within 5 kb of the center of the TSS (Transcription Start Sites) region of the 2C-regulated genes. (E) Relative gene expression normalized to *Gapdh* upon indicated manipulation. P values were calculated by unpaired t-test, two-tailed, two-sample unequal variance, ns = no significance, ** < 0.01, *** < 0.001, **** < 0.0001. (F) A box plot showing the $\log_2$ FC (fold change) of Zfp281-unbound-2C-upregulated transcripts in Zfp281-perturbed and control mESCs after *Dux* induction. The black central line is the median, the box limits indicate the upper and lower quartiles. P values were calculated by the Wilcoxon rank-sum test unless otherwise stated, ** < 0.01. The dots represent outliers. (A-F) Doxycycline (Dox) was used at 2µg/µl; the X in sgX refers to the gene that sgRNA targets to; sgSv40 is negative control.

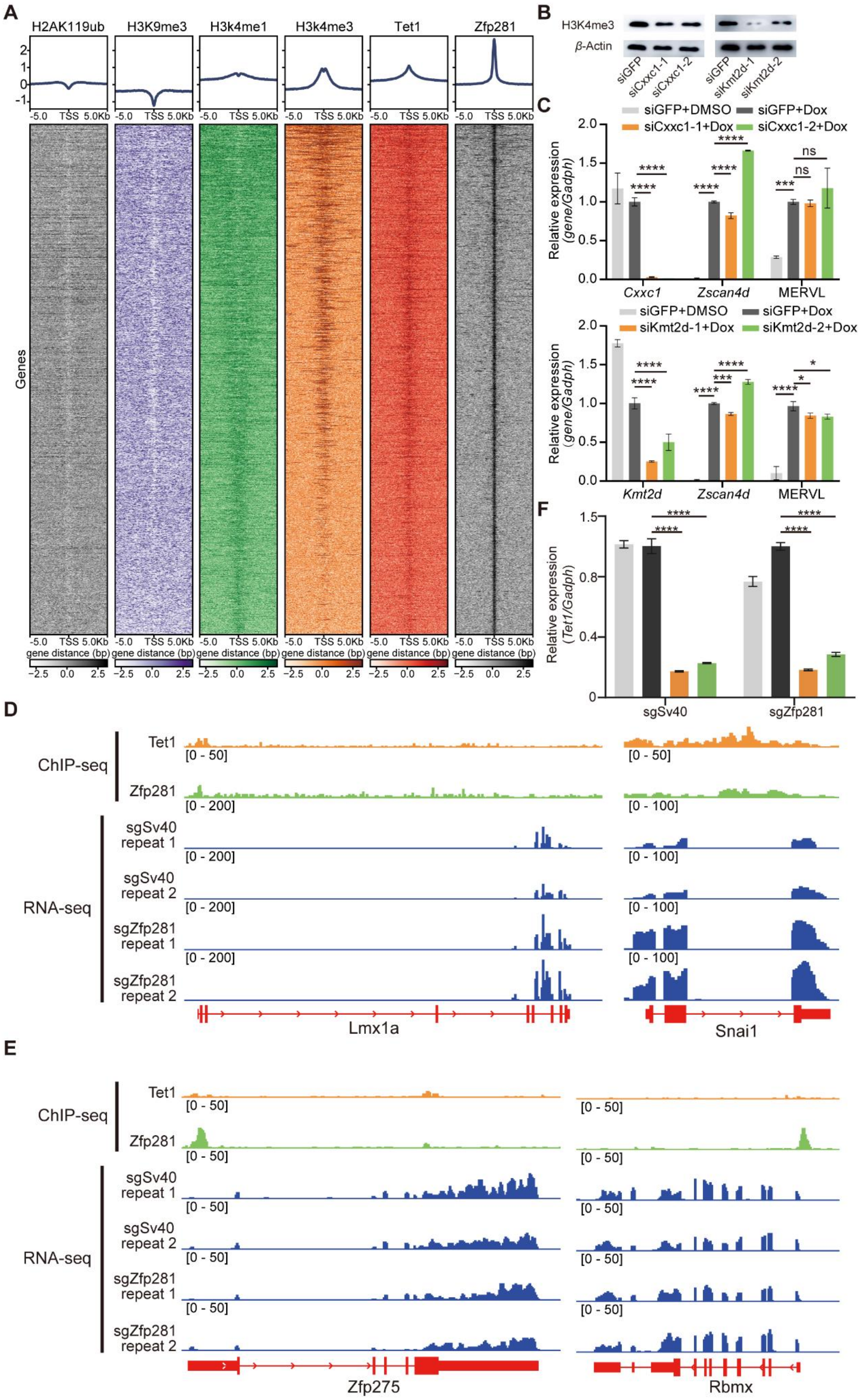
A
H2AK119ub
H3K9me3
H3k4me1
H3k4me3
Tet1
Zfp281
-5.0 TSS 5.0Kb
Genes
gene distance (bp)
B
H3K4me3
β-Actin
siGFP
siCxxc1-1
siCxxc1-2
siKmt2d-1
siKmt2d-2
C
siGFP+DMSO
siGFP+Dox
siCxxc1-1+Dox
siCxxc1-2+Dox
Relative expression (gene/Gadph)
Cxxc1
Zscan4d
MERVL
ns
siKmt2d-1+Dox
siKmt2d-2+Dox
Kmt2d
F
Relative expression (Tet1/Gadph)
sgSv40
sgZfp281
D
ChIP-seq
RNA-seq
Tet1
Zfp281
sgSv40 repeat 1
sgSv40 repeat 2
sgZfp281 repeat 1
sgZfp281 repeat 2
[0 - 50]
[0 - 200]
[0 - 100]
Lmx1a
Snai1
E
Zfp275
Rbmx

44

Fig. S3, Tet1 mediates the transcriptional regulation of Zfp281 on 2C-regulated genes. (A) Average occupancy plots and heatmaps of the binding profiles in mESCs for Zfp281, Tet1, H2AK119ub, H3K9me3, H3K4me1, and H3K4me3 within 5 kb of the center of the Zfp281 peaks are shown. The exact same genomic regions are shown in each heatmap. (B) Western blot of H3K4me3 upon indicated manipulation in mESCs. (C) Relative mRNA levels of *Cxxc1*, *Kmt2d*, *Zscan4d*, and MERVL normalized to *Gadph* in mESCs in mESCs upon indicated manipulation. Dox represents doxycycline. (D) Expression levels of *Tet1* normalized to Gadph in mESCs upon indicated manipulation. (D) RNA-seq and ChIP-seq genome browser track showing increased expression of the *Lmx1a* and *Snail1* (Zfp281 and Tet1-cobound genes) upon Zfp281 perturbation in Dux-activated mESCs. (E) RNA-seq and ChIP-seq genome browser track showing unchanged expression of the *Rbmx* and *Zfp275* (Zfp281-bound Tet1-unbound genes) upon Zfp281 perturbation in Dux-activated mESCs. The RNA-seq results are displayed as reads per kilobase million. (B-E) Doxycycline (Dox) was used at 2μg/μl; the X in siX/sgX refers to the gene that siRNA/sgRNA targets; siGFP and sgSv40 are the negative control. Shown are mean ± s.d, n = 3. P values were calculated by the parametric one-way analysis of variance (ANOVA) test, ns = no significance, ** < 0.01, *** < 0.001, **** < 0.0001.

Supplementary Table

Table S1. Primers for real-time PCR

Gene	Sequence (5'-3')
Tet1	F:- ACACAGTGGTGCTAATGCAG-<br>R:- AGCATGAACGGGAGAATCGG-
Cxxc1	F:- TTGGATGTGACAACTGCAACG-<br>R:- GTGGCGGTAACGAATCTCCAG-
Kmt2d	F:- GTGGCTGTTCCACACCCAG-<br>R:- AGCTTGAGCTTCTCAGCATCG-
MERVL	F:-CTCTACCACTTGGACCATATGAC-<br>R:-GAGGCTCCAAACAGCATCTCTA-
Zfp352	F:-AGAGGACAAGACCCAGTGCAG-<br>R:-GAGGTCCTCATCTGACCCAAG-
Gapdh	F:-CATGGCCTTCCGTGTTCCTA-<br>R:-GCCTGCTTACCACCTTCTT-
Zscan4d	F:-AAATGCCTTATGTCTGTTCCCTATG-<br>R:-TGTGGTAATTCCTCAGGTGACGAT-
syn*Dux*	F:-TCACAACCCCCACAAGTGG-<br>R:-TCCTCCAACTGTACTTCAGTAA-
Dux	F:-CAACGCAGGCTCTATGGAAC-<br>R:-GCTTCTTCCTGTGGCCAAAA-

Table S2. The sequence of the sgRNA and siRNA for Zfp281

Gene	Sequence (5'-3')
Zfp281-1	GAGGATAACACGCACTGCGG
Zfp281-2	GACTGATGAGCACGGCAACC
siZfp281-1	ACCTTTATCACTAACTCTAAT
siZfp281-2	GAGCCAAAGCAGGACGCTAAC
